# LACE Score-Based Risk Management Tool for Long-Term Home Care Patients: A Proof-of-Concept Study in Taiwan

**DOI:** 10.3390/ijerph18031135

**Published:** 2021-01-28

**Authors:** Mei-Chin Su, Yu-Chun Chen, Mei-Shu Huang, Yen-Hsi Lin, Li-Hwa Lin, Hsiao-Ting Chang, Tzeng-Ji Chen

**Affiliations:** 1 Department of Nursing, Taipei Veterans General Hospital, Taipei 112, Taiwan; mcsu@vghtpe.gov.tw (M.-C.S.); huangms@vghtpe.gov.tw (M.-S.H.); lhlin@vghtpe.gov.tw (L.-H.L.); 2 Institute of Hospital and Health Care Administration, National Yang Ming Chiao Tung University, Taipei 112, Taiwan; tjchen@vghtpe.gov.tw; 3 Department of Family Medicine, Taipei Veterans General Hospital, Taipei 112, Taiwan; sonia1999082727@yahoo.com (Y.-H.L.); htchang2@vghtpe.gov.tw (H.-T.C.); 4 School of Medicine, National Yang Ming Chiao Tung University, Taipei 112, Taiwan; 5 Institute of Public Health, National Yang Ming Chiao Tung University, Taipei 112, Taiwan

**Keywords:** long-term care (LTC), long-term home care (LTHC), readmission, LACE score, predictive model, readmission risk management

## Abstract

Background: Effectively predicting and reducing readmission in long-term home care (LTHC) is challenging. We proposed, validated, and evaluated a risk management tool that stratifies LTHC patients by LACE predictive score for readmission risk, which can further help home care providers intervene with individualized preventive plans. Method: A before-and-after study was conducted by a LTHC unit in Taiwan. Patients with acute hospitalization within 30 days after discharge in the unit were enrolled as two cohorts (Pre-Implement cohort in 2017 and Post-Implement cohort in 2019). LACE score performance was evaluated by calibration and discrimination (AUC, area under receiver operator characteristic (ROC) curve). The clinical utility was evaluated by negative predictive value (NPV). Results: There were 48 patients with 87 acute hospitalizations in Pre-Implement cohort, and 132 patients with 179 hospitalizations in Post-Implement cohort. These LTHC patients were of older age, mostly intubated, and had more comorbidities. There was a significant reduction in readmission rate by 44.7% (readmission rate 25.3% vs. 14.0% in both cohorts). Although LACE score predictive model still has room for improvement (AUC = 0.598), it showed the potential as a useful screening tool (NPV, 87.9%; 95% C.I., 74.2–94.8). The reduction effect is more pronounced in infection-related readmission. Conclusion: As real-world evidence, LACE score-based risk management tool significantly reduced readmission by 44.7% in this LTHC unit. Larger scale studies involving multiple homecare units are needed to assess the generalizability of this study.

## 1. Introduction

Discharging long-term home care (LTHC) patients, i.e., long-term care (LTC) patients at home, from the hospital is an intricate process and challenging [1,2,3,4]. Considerable research attention has been devoted to risk management for readmission reduction to avoid excess mortality and medical cost [1,4,5]. In general, readmission reduction programs consist of two major components: stratifying patients by readmission risk and setting-up individualized preventive plans [6]. However, most readmission reduction programs usually work specifically for certain clinical condition [7]. Since LTHC patients were usually at older age with multiple comorbidities, it is challenging to balance between investments in a readmission reduction effort and readmission cost for homecare providers [8,9]. Risk management predictive models allow home care providers to arrange the priority of medical interventions according to the patient’s hospitalization risk and optimize the utilization of medical resources [10]. However, there remains a gap in putting predictive models into actual practice for home care patients [11].

LACE score is a readmission predictive model for various clinical scenarios including inpatients [12], surgical patients [13], chronic obstructive pulmonary disease (COPD) [14], and heart failure patients [15]. Preventive intervention can be prioritized according to patients’ risk [2,10,13,16,17,18]. Due to its simplicity features, LACE score gained attention among many emerging readmission predictive models [19]. According to LACE model, the probability of a patient’s readmission risk can be ideally calculated by four components, including the length of stay (L), acute admission (A), comorbidity (C), and emergency department visits in the previous six months (E) so that home care providers can stratify the patients and directed resource to the patients in need [20]. Although LACE score has been used in quite a few scenarios, it has been criticized for the poor performance in certain conditions [21,22,23]. The clinical utility of LACE score for risk management in LTHC patients remains unanswered.

In Taiwan, home care visits are specifically reimbursed toward patients who are bed- or wheel-chair bounded and severely dependent in activities of daily living (ADL) by Taiwan’s National Health Insurance (NHI) system [24,25]. As home care services are under government payment, the health system would be heavily impacted by home care patients’ readmission [11,26]. As a result, a novel home care delivery method, LACE score-based readmission risk management tool had been proposed and simulated in our home care setting previously [20]. While there has been a great deal of research on predictive models and readmission reduction, very few studies focus on integrating predictive models into actual practice for LTHC patients. This study reports a real-world experience in using LACE score-based readmission risk management tool to reduce readmission in LTHC patients. The aim of this study is twofold. The first is to validate LACE score in our home care setting. The second is to assess the effect of LACE score-based risk management tool. Our results should be a reference for quality improvement for home care services.

## 2. Materials and Methods

### 2.1. Setting, Data Source, and Ethical Concerns

A before-and-after study was conducted among LTHC patients managed by one medical center affiliated home care unit in Taipei, Taiwan. This home care unit features a service volume of 150–240 patients per year and an elderly patient population (i.e., age 80–88 years on average in past years). Home care patients are mostly diagnosed with cerebrovascular disease, hypertension, or diabetes. They are not only totally or severely dependent in ADL (i.e., Barthel index < 50) but 93–95% of them also have nasogastric tubes, urinary tubes, or tracheal tubes [25]. Referrals from its affiliated medical center contribute to 95–98% of these patients. Using the hospital’s electronic medical data (EMR), demographic information, and medical records of these patients were obtained. Age, gender, and all variables as predicting factors of LACE score were included.

This study’s protocol was approved by the Institutional Review Board of Taipei Veterans General Hospital (IRB #2018-08-016AC). The Institutional Review Board waived the requirement of written informed consent from each patient involved in our study because the data we analyzed was de-identified. Moreover, no identifiable private information or human biospecimens was involved.

### 2.2. Pre-Implement Cohort vs. Post-Implement Cohort

**Figure 1:**
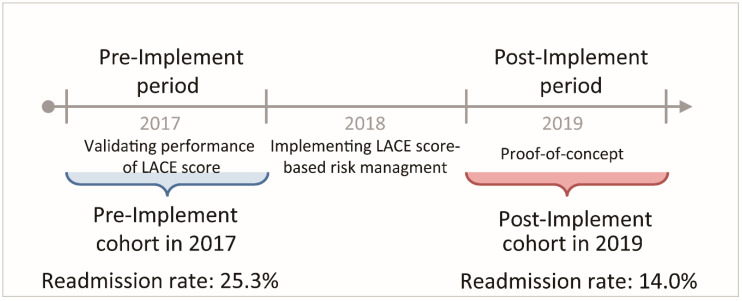
Definition of study cohorts before and after implementing LACE score-based predictive risk management tool in the medical center affiliated home care unit in Taiwan, 2017 and 2019.

A before-after study design was used to compare the effect of LACE score-based risk management tool. To validate the performance of LACE score and collect baseline information, we enrolled LTHC patients who had had any acute hospitalization between 1 January 2017 and 31 December 2017 as the Pre-Implement cohort in 2017. Moreover, we enrolled patients who had been hospitalized between 1 January 2019 and 31 December 2019 as the Post-Implement cohort in 2019 to evaluate the risk reduction effect of risk management tool. The one-year gap (2018) of Pre-Implement cohort and Post-Implement cohort was used to minimize the bias introduced by clinical providers’ learning and training time lag (Figure 1).

For both groups of cohorts, admissions with a length of stays of more than 24 h were included, while shorter ones were excluded to avoid including observation stays. We also excluded admissions with certain events: death during hospitalization, a patient being transferred to another acute health care facility or chronic health care facility (e.g., nursing home and respite care center) after discharge, or patient discharged against medical advice. For multiple hospitalizations made by a single patient, variables of each of them are counted on an admission-by-admission basis.

### 2.3. Definition of 30-Day Readmission, Readmission Rate, and Cause-Specific Readmission Rates

The EMR in the hospital provided a very comprehensive follow-up in this study. Every acute hospitalization in the study cohorts is follow-up for 30 days after discharge. Hospitalizations are regarded as “hospitalization with 30-day readmissions” if patients are readmitted within 30 days after their discharge, whereas hospitalizations are regarded as “hospitalization with successful discharge” if patients are free from readmission. Readmission rates are calculated as the number of “hospitalization with 30-day readmissions” divided by the number of hospitalizations. Cause-specific readmission rates are calculated as the number of acute hospitalizations (readmission) within 30 days with specific cause for hospitalization divided by the total number of acute hospitalizations. Multiple eligible hospitalizations made by a single patient are counted separately. The bias of lost follow-up should be minimal because the home care patients who participated in this study were usually sent back to the same hospital as the home care unit if it were necessary.

### 2.4. Calculation of LACE Score

The LACE scores are calculated by points assigned for the patient’s length of stay, the acuity of the patient’s admission, the degree of comorbid illness or illnesses (as measured by the Charlson comorbidity index), and the number of times patients had been to the emergency department in the last six months. The higher the patients’ LACE scores are, the more chances for them to get readmitted (Appendix A Table A1) [19]. We carefully define the four components of LACE score with the best compatibility with the original LACE score definitions as the following: [19]

#### 2.4.1. L: Length of Stay

Length of stay is the calculation of days from the first day of hospitalization to the last day. For readmission that occurs on the same day as the discharge is considered the same hospitalization.

#### 2.4.2. A: Acute Admission

Patients admitted to the medical center via the emergency department is identified as acute admissions in contrast to planned admissions.

#### 2.4.3. C: Comorbidity

Comorbidities are measured by the Charlson comorbidity index (CCI) score [19]. The primary or secondary diagnostic International Classification of Diseases, Ninth Revision (ICD-9) in the previous year of the index admission was used in calculating the CCI score [20].

#### 2.4.4. E: Emergency Department Visits in the Previous Six Months

The number of visits of each patient is measured. However, multiple emergency visits within 24 h are considered the same visit.

### 2.5. LACE Score-Based Risk Management Tool for Readmission Reduction

In Taiwan, home care visits are reimbursed specific to LTHC patients who are totally or severely disabled. Since only two home visits per month are covered by Taiwan’s National Health Insurance system, home care units must balance the operational cost and payment to maintain its sustainability. As a result, before 2018, most home visits of the home care unit were scheduled periodically and commonly with a standard follow-up protocol.

Started from 2018, we implemented LACE score-based risk management tool and made an internal consensus on the readmission prevention program. Every home care patient’s LACE score was calculated after their acute hospitalization. Once a patient was identified as high-risk for readmission, i.e., LACE score higher than the cut-off value, home care physician and home care nurse would plan an individualized readmission reduction plan for him/her in addition to the standard follow-up [19,27]. The responsible home care nurse would make telephone calls to assess and initiate a readmission prevention plan within 72 h immediately after their discharge. The readmission prevention plan was modified from the BOOST (Better Outcomes for Older adults through Safe Transitions) tool [28] and it basically included the following components: (1) Identifying: past admission history was rigorously reviewed for the reason for admission. (2) Supporting: Follow-up phone call at 72 h to assess the condition, adherence, and complications; a follow-up home visit within seven days of hospital discharge; build link to community resource if needed. (3) Prevention: disease-specific education; action plan regarding what to do and whom to contact for worsening or new symptoms; drug education especially for antibiotics. A demonstration and return demonstration are carried out to ensure that the caregiver knows essential knowledge, attitude, and techniques for their own readmission reduction plan.

### 2.6. Statistical Analysis

Statistical analyses were performed using IBM SPSS Statistics, version 26, (I.B.M. Corp., Armonk, NY, USA) and MedCalc, version 19 (MedCalc Software, Ostend, Belgium). The study population was described by age, gender, and all the components of LACE score. Univariate analysis was performed by Chi-square test and a t-test to evaluate similarities between the Pre-Implement cohort in 2017 and the Post-Implement cohort in 2019.

The performance of LACE score of the Pre-Implement cohort in 2017 was evaluated in terms of calibration, using the Hosmer–Lemeshow test to test the closeness of predicted probability to observed probability, and in terms of discrimination, using the receiver operator characteristic (ROC) curve and the area under ROC curve (AUC) to test the ability of LACE score to separate patients by their risk of readmission. It is suggested that an AUC less than 0.60 reflects poor discrimination; 0.60 to 0.75, possibly helpful discrimination; and more than 0.75, clearly useful [27]. The Youden index was used to obtain the optimal cut-off value of LACE score for readmission. The clinical utility was evaluated in terms of positive predictive value (PPV), positive likelihood ratio (+LR), negative predictive value (NPV), and negative likelihood ratio (-LR) at the cut-off value; 95% confidence intervals were estimated based on binominal distribution.

To assess the effectiveness after implementing LACE score-based risk management tool, 30-day readmission, cause-specific readmission rates, and rate differences of Pre-Implement cohort in 2017 and Post-Implement cohort in 2019 were calculated. Two-sided *p*-values < 0.05 were considered significant.

## 3. Results

### 3.1. Pre-Implement Cohort and Post-Implement Cohort

During the first year of the study period, before implementing LACE score-based predictive risk management tool for readmission in 2017, 161 LTHC patients of the home care unit were identified, and there were 48 patients with a total of 87 acute hospitalizations (overall admission rate 54.0%) enrolled as the Pre-Implement cohort for validating purpose. In 2019, one year after implementing LACE score-based predictive risk management tool for readmission, there were 361 patients of the home care unit, and there were 132 patients with 179 acute hospitalizations (overall admission rate 49.6%) enrolled as the Post-Implement cohort for the purpose of proof-of-concept (Table 1).

Pre-Implement and Post-Implement cohorts were similar in terms of demographics and disease severity, while there was a 1.24-fold increase in service amount in 2019. In 2017 and 2019, the ratio of male to female cohorts is close to 1:1, with an average age of about 80 years old (*p* = 0.711 for gender; *p* = 0.094 for age). The average LACE score in both two groups was about 11 (*p* = 0.465), which means the population composition, the severity of illness, and the risk of hospital readmission were equal in the two groups. We can compare the outcome with minimal confounding bias (Table 1).

### 3.2. Validating Performance of LACE Score

**Figure 2:**
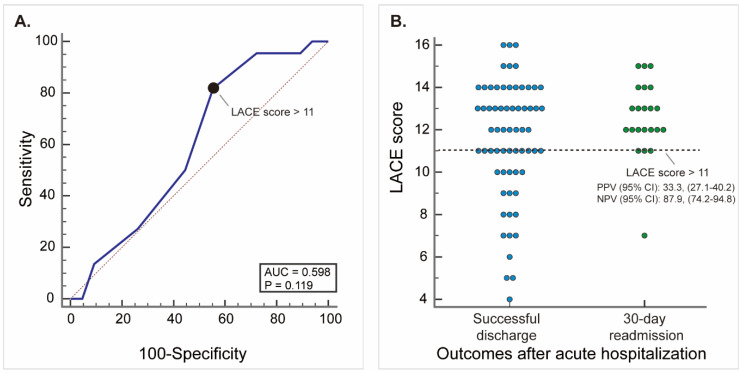
(**A**) Receiver operator characteristic (ROC) curve of validating LACE score for readmission in the Pre-Implement cohort in the long-term home care unit in 2017 (*n* = 87, 2017). The cut-off value based on the Youden index is also shown as the solid black circle in the chart. (**B**) Distribution of LACE score for readmission in patients with successful discharge and 30-day readmission. Each dot represents one patient with its color denotes a different outcome. The horizontal line in the chart shows the cut-off value based on the Youden index. (The Pre-management cohort in 2017, *n* = 87).

Although LACE score predictive model only showed an acceptable performance result, it did show the potential as a useful screening tool for risk management for home care patients. With respect to the performance of the LACE score for readmission risk in the Pre-Implement cohort in 2017, the calibration and discrimination results are shown in Figure 2.

LACE score fitted well in the Pre-Implement cohort in 2017. The prediction of probabilities for readmission rates was close to the actual readmission rates (Hosmer–Lemeshow test, *p* = 0.171). However, the discrimination ability of LACE score was poor. In the Pre-Implement cohort in 2017, AUC is 0.598 (95% C.I., 0.488–0.702), the ROC curve in the upper right region seems good (sensitivity between 50 and 100) but the concavity of the ROC curve disappeared in the left lower region in the lower left region (sensitivity between 0 and 50) (Figure 2A). This finding suggested that LACE score might provide a good readmission prediction for certain patients but could not accurately determine the readmission for every home care patient. The LACE score’s optimal cut-off value was determined by Youden index, and patients whose LACE score were higher than 11 were considered high risk for readmission (Figure 2A).

LACE score had good potential in stratifying patients by their risk for readmission. For the given cut-off value (LACE score >11), LACE score showed a high sensitivity at 81.82% (95% C.I., 59.7–94.8) with a low specificity at 44.62% (95% C.I., 32.3–57.5). NPV is 87.9% (95% C.I., 74.2–94.8), which means that 87.9% of patients who were stratified as low-risk were correct (successfully discharge without readmission). PPV is 33.3% (95% C.I., 27.1–40.2) which means only one-third of high-risk patients actually readmitted. +LR is 1.48 (95% C.I., 1.1–2.0) and -LR is 0.41 (95% C.I., 0.2–1.0) means that LACE score is more powerful in ruling-out disease than ruling-in disease. As a result, Figure 2B showed that LACE score (the dotted line) clearly separated home care patients into high-risk (the upper part above the dotted line) and low-risk (the lower part below the dotted line), which suggested its value of a screening tool (ruling-out disease) for readmission.

### 3.3. Readmissions before and after Implementing LACE Score-Based Predictive Risk Management Tool

Implementing LACE score-based predictive risk management tool did reduce readmission. Once a patient was identified with a high risk for readmission, home care staff could promptly contact the patient within 72 h after discharge. A risk reduction plan would be initiated immediately in addition to the standard care protocol. As a result, there was a significant reduction of 44.7% in readmission rate in the Post-Implement cohort in 2019 (readmission rate, 14.0%; 95% C.I., 9.0–20.3), compared to that in the Pre-Implement cohort in 2017 (readmission rate, 25.3%; 95% C.I., 15.9–38.3) (rate difference, 11.32%; 95% C.I., 0.6–22.1, *p* = 0.0393) (Table 2).

**Figure 3:**
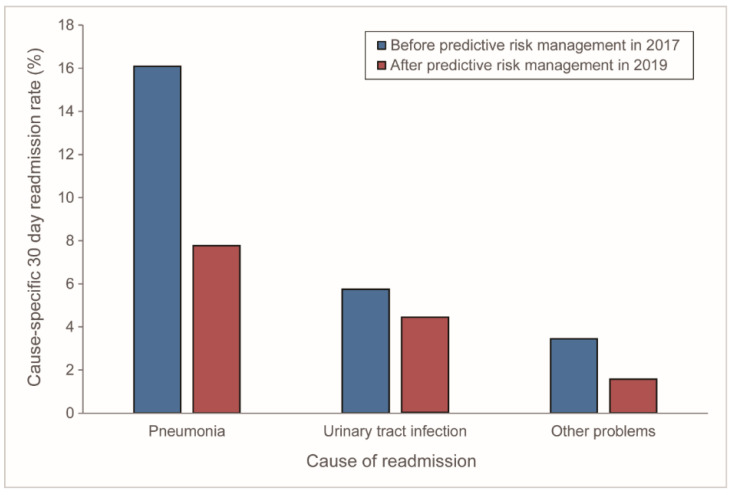
Cause-specific readmission rates by causes of readmission before (Pre-Implement) and after (Post-Implement) implementing LACE score-based predictive risk management tool to long-term home care patients of a medical center affiliated home care unit in Taiwan in 2017 and 2019. (Pre-Implement cohort in 2017, *n* = 87; Post-Implement cohort in 2019, *n* = 179).

LTHC patients were frequently readmitted because of infection problems. After implementing LACE score-based predictive risk management tool, the infection-specific readmission rate effectively decreased from 24.1% in 2017 to 12.3% in 2019 (readmission rate difference, 11.9%, 95% C.I., 1.6–22.5, *p* = 0.02) (Appendix A Table A2 and Table A3). There were an 11.3% reduction in readmission rate for pneumonia, and an 8.3% reduction for urinary tract infection in the Post-Implement cohort in 2019 compared to that in the Pre-Implement cohort in 2017 (Table 2; Figure 3). These findings suggested that the implementation of LACE score-based predictive risk management tool can lower the readmission rate for LTHC patients, specifically for those with infection-related problems.

## 4. Discussion

LTHC patients are vulnerable to readmission; however, it is challenging to effectively reducing readmission. We provided unique real-world experience in the implementation of LACE score-based readmission risk management tool for LTHC patients. The current before-and-after study validated LACE score, a predictive model for readmission, and evaluated the effect after implementing LACE score-based risk management tool. Although LACE score predictive model (AUC = 0.598) still has room for improvement, it did show the potential as a good screening tool for risk management for LTHC patients (NPV, 87.9%; 95% C.I., 74.2–94.8; -LR, 0.41; 95% C.I., 0.2–1.0). The result showed that LACE score-based predictive risk management tool could greatly reduce readmission in LTHC patients. For LTHC patients, the readmission rate of Post-Implement cohort in 2019 was significantly reduced by 44.7% (rate difference, 11.32%; 95% C.I., 0.6–22.1, *p* = 0.0393). This study provides an exceptional real-world experience in using LACE score-based risk management tool to reduce readmission rates among LTHC patients. The reduction effect is more pronounced in infection-related readmission. However, the result applies only to the current LTHC setting and requires further validations.

Our results filled the gap in understanding how LACE score-based risk management tool reduces readmission in LTHC patients. Our study showed that LTHC patients could be stratified by LACE scores, and a subsequent individualized preventive plans for those in needed on time had clearly cut down readmission rates [6]. Unlike previous studies, the current study further provided a convincing evidence that our tool works well for LTHC patients who were severely or totally dependent in ADL with older age, mostly intubated with nasogastric tubes, urinary catheters, and tracheal tubes, and had more comorbidities. Since LTHC patients are much different from other home care populations with better functional status, the infection-related problem rises to the leading and majority reason for readmission instead of traditional drivers of readmissions such as COPD, heart failure, and acute myocardial infarction [29].

Although some believe that LACE score model cannot perform well in older adults, our result shows its clinical usefulness in readmission risk management for LTHC patients. The clinical value of a prediction model cannot be determined solely by discrimination (i.e., AUC) and calibration [27] as the previous studies did [21,22,23]. In home care settings, there is an ethical consideration for false negatives (no preventive measure for high-risk patients predicted as low-risk) and false positives. (overdone preventive measure for low-risk patients predicted as high-risk) [30] As a result, ruling-out readmission is much more critical than ruling-in readmission in home care patients [31]. Since readmission reduction plan takes extra cost to the standard follow-up, ruling-out readmission (higher NPV and -LR, higher power to identify patients had a lower risk for readmission) can save the excessive cost of additional preventive measure to low-risk patients, and direct resources to high-risk ones [32,33]. This finding is compatible with our previous simulation [20], and this is likely why there is still room for improvement for LACE score, but a substantial reduction in readmission rate.

Infection-related problems are crucial for readmission for home care patients, and LACE score-based risk management tool may address this issue. Since home care patients are more likely to develop infection than the general population, the caregivers’ training in providing needed care at home is essential [34]. By using LACE score, home care nurses can straightly identify the high-risk patients and the families who need help. Nurses can immediately direct additional infection control intervention resources to high-risk patients and educate them based on patients’ health literacy level to complete understanding [35]. Our findings are consistent with previous results showing the effectiveness of readmission risk management [36].

Immediate and efficient outpatient communication are the keys to a successful readmission reduction plan. A nurse-led, immediate, and intensive outpatient communication can identify developing problems and decrease unplanned hospital readmission [37]. Like the previous study, nurse-led motivational and timely telephone follow-up can positively influence self-management and reduce hospital readmission [38], while a majority of readmission occurred within seven days right after discharge [29]. However, the increasing cost of intensive communication can only be resolved by either change in services-delivery or technology-assisted services [26]. In our experience, LACE score-based risk management tool considerably changed the way of regular-visit-based home visits. It provided home care nurses with information to effectively and efficiently schedule their home visits according to patients’ risk [38,39]. Moreover, our result encouraged future studies on telehealth to increase the efficiency of home visit. Examples such as MonashWatch in Australia had successfully reduced length of stay with high patient satisfaction should be a scalable solution for home care [40]. While a majority of health services had been lockdown recently because of the pandemic of COVID-19, there is a surge of using telehealth, e.g., a 154% increase in the United States [41]. Using telehealth including remote monitoring, televisit, telenursing, telenutrition, telepharmacy, and telerehabilitation should greatly support LTHC patients [42].

Although there is room for improvement of LACE score, a trade-off between simplicity and accuracy is evident. There is an emerging trend of developing predictive models. By introducing abundant variables in EMR database [43], collecting social determinants of health [44], and using sophisticated machine learning methods can build a surprisingly accurate but complex readmission prediction model [2,16,45,46]. There is always a huge information gap between the transition from hospital to home, and missing data are not uncommon for home care patients. This missingness would make the models above resulted in suboptimal predictions [47,48]. Our experience showed that LACE score is useful for LTHC patients in our home care unit since LACE score can be easily calculated by only four routinely collected variables. However, we encourage a system-level integration of clinical data to bridge the information gap between each transition. The more clinical data available, the more prediction models can be practically useful for efficiency and quality improvement in home care [10,11].

Our study had some limitations. First, our study focused on LTHC patients, a particular group of disabled patients with a high percentage of nasogastric tubes, urinary catheters, or tracheostomy tubes; thus, our result may not apply to a general home care population. Moreover, readmission rates in the current study should be adjusted when comparing to those in other studies [49]. Second, our findings are not generalizable beyond the study setting. As a single medical center and its home care unit, we may have better medical and logistic support than other home care providers. As a result, our result might be over-optimistic. A large scale and multiple center study are warranted. Third, the current study used a before-and-after study design, time effect, and observer bias should have existed. These biases might inevitably over-estimate the effect of the risk reduction plan. We encouraged a long-term follow-up study and aggregation of evidence from multiple home care units to examine the effect of risk management tool. Fourth, though the EMR database provided comprehensive information intra-hospital, we could not recognize the admissions and readmissions outside of this hospital of our study. The missingness might slightly underestimate admission rates in both groups. However, such missing bias should be minimal since the home care patients who participated in this study were usually sent back to the same hospital as the home care unit. Fifth, we did not adjudicate whether each readmission was elective versus unplanned. However, based on our experience, we would expect the rate of elective readmissions on home care services to be low. These limitations might reduce the generalizability of our results.

## 5. Conclusions

As real-world evidence, LACE score-based risk management tool significantly reduced readmission by 44.7% for LTHC patients in our home care unit, and the effect is more pronounced for infection-related problems. There is room for improvement in LACE predictive score’s predictive power, and larger scale, multiple homecare unit studies will have to assess the generalizability of our study.

## Figures and Tables

**Table 1:** Demographic information, LACE score, and outcome before (Pre-Implement) and after (Post-Implement) implementing LACE score-based predictive risk management tool to long-term home care (LTHC) patients a medical center affiliated home care unit in Taiwan in 2017 and 2019. (Pre-Implement cohort in 2017, *n* = 87; Post-Implement cohort in 2019, *n* = 179).

	Acute Hospitalizations of the Pre-Implement Cohort in 2017 (*n* = 87)		Acute Hospitalizations of the Post-Implement Cohort in 2019 (*n* = 179)			
	Mean	(SD ^1^)	Mean	(SD ^1^)	*p*-Value	Sig. ^2^
Personal Demographics						
Gender					0.711	
Female, no. (%)	40	(46.0)	78	(43.6)		
Male, no. (%)	47	(54.0)	101	(56.4)		
Age	87.4	(12.4)	84.7	(12.0)	0.094	
LACE Score						
Length of stay (L)	5.6	(0.7)	5.7	(0.6)	0.229	
Acuity of admission (A)	2.7	(0.9)	2.8	(0.6)	0.283	
Comorbidities (C)	1.7	(0.9)	1.6	(0.9)	0.396	
Emergency department visits (E)	1.9	(1.3)	1.8	(1.1)	0.513	
LACE score	11.8	(2.6)	11.6	(1.8)	0.465	
Outcome						
No. of 30-day readmission	22		25		0.023	*
Readmission rate %, (95% C.I. ^3^)	25.3	(15.9–38.3)	14.0	(9.0–20.3)	0.039	*

^1^ SD: standard deviation; ^2^ Sig.: significance level: *p*-Value <0.05: *; ^3^ 95% C.I.: 95% confidence interval.

**Table 2:** 30-day readmissions and cause-specific readmission rates before (Pre-Implement) and after (Post-Implement) implementing LACE score-based predictive risk management tool to long-term home care patients of a medical center affiliated home care unit in Taiwan in 2017 and 2019. (Pre-Implement cohort in 2017, *n* = 87; Post-Implement cohort in 2019, *n* = 179).

Causes of Readmission	Readmissions before Predictive Risk Management in 2017 ^1^ (Pre-Implement in 2017, *n* = 87)		Readmissions after Predictive Risk Management in 2019 ^2^ (Post-Implement in 2019, *n* = 179)		Readmission Rate Difference (%)
	No. of Readmissions	Cause-Specific Readmission Rate (%) ^3^	No. of Readmissions	Cause-Specific Readmission Rate (%) ^3^	
All-cause	22	25.3	25	14.0	11.3
Pneumonia	14	16.1	14	7.8	8.3
Urinary tract infection	5	5.7	8	4.5	1.3
Other problems	3	3.4	3	1.7	1.7

^1^ The total number of acute hospitalizations in the home care unit before implementing LACE score-based predictive risk management tool in 2017 is 87. ^2^ The total number of acute hospitalizations after implementing LACE score-based predictive risk management tool in 2019 is 179 because of the increasing service amount of the home care unit. ^3^ Cause-specific readmission rates were calculated as the number of acute hospitalizations (readmission) within 30 days following previous acute hospitalization of each cause of readmission divided by the total number of acute hospitalizations.

## Appendix A

**Table A1:** Components of LACE score [19].

Attribute	Value	Points
Length of stay (days)	<1	0
	1	1
	2	2
	3	3
	4–6	4
	7–13	5
	≥14	7
Acute admission	Yes	3
Comorbidity: Charlson comorbidity index score	0	0
	1	1
	2	2
	3	3
	≥4	5
Emergency department visits in previous 6 months	0	0
	1	1
	2	2
	3	3
	≥4	4

**Table A2:** Readmissions and cause-specific readmission rate before implementing LACE score-based predictive risk management tool to a home care patient cohort of a medical center affiliated home care unit in Taiwan in 2017. (*n* = 87, 2017).

Causes of Readmission	No. of Readmissions	Cause-Specific Readmission Rate (%)
Pneumonia	14	16.1
Urinary tract infection	5	5.7
Herpes zoster infection	1	1.1
Gastrointestinal ulcers	1	1.1
Acute myocardial infarction	1	1.1

**Table A3:** Readmissions and cause-specific readmission rate after implementing LACE score-based predictive risk management tool to a home care patient cohort of a medical center affiliated home care unit in Taiwan in 2019. (*n* = 190, 2019).

Causes of Readmission	No. of Readmissions	Cause-Specific Readmission Rate (%)
Pneumonia	14	7.4
Urinary tract infection	8	4.2
Fever of unknown origin	2	1.1
Gastrointestinal ulcers	1	0.5
